# Functional outcomes and quality of life following free fibula flap harvest: a comparative analysis of flexor hallucis longus resection versus preservation

**DOI:** 10.3389/fonc.2025.1651547

**Published:** 2025-09-05

**Authors:** Guanghui Zhao, Xu Zhang, Wei Du, Junhui Yuan, Qigen Fang

**Affiliations:** ^1^ Department of Spine Surgery, Zhumadian Central Hospital, Zhumadian, China; ^2^ Department of Head Neck and Thyroid, The Affiliated Cancer Hospital of Zhengzhou University & Henan Cancer Hospital, Zhengzhou, China; ^3^ Department of Radiology, The Affiliated Cancer Hospital of Zhengzhou University & Henan Cancer Hospital, Zhengzhou, China

**Keywords:** free fibula flap, quality of Life, flexor hallucis longus, donor site morbidity, objective assessment

## Abstract

**Background:**

The free fibula flap (FFF) is a gold standard for maxillofacial reconstruction, yet debate persists regarding the functional impact of flexor hallucis longus (FHL) resection. This study evaluates donor-site morbidity and quality of life (QoL) following FFF reconstruction, comparing outcomes with and without FHL resection.

**Methods:**

A retrospective analysis of prospectively collected data was conducted for 93 patients undergoing FFF reconstruction. Patients were stratified into FHL-harvested and non-FHL groups. Primary outcomes included QoL, AOFAS scores, hallux flexion strength, range of motion (ROM), isokinetic dynamometry, and gait analysis. Assessments were performed preoperatively and at 3- and 6-month intervals.

**Results:**

The study cohort comprised 93 patients, with FHL harvested in 43 cases. The FHL group exhibited transient declines in QoL at 3 months, with partial recovery by 6 months. Both groups showed similar AOFAS score trajectories, with temporary declines at 3 months and near-complete recovery by 6 months. Hallux flexion strength decreased comparably in both groups at 3 months (FHL: −38%; non-FHL: −34%), with residual 18–20% deficits at 6 months. Isokinetic testing revealed transient plantar flexion weakness in the FHL group at higher velocities (90°/s: 54.2 ± 7.0 Nm vs. 58.6 ± 7.5 Nm pre-op, p=0.008), resolving by 6 months. Gait analysis demonstrated initial impairments in stride length and ankle ROM in the FHL group, normalizing by 6 months.

**Conclusion:**

FHL resection during FFF harvest leads to transient functional and QoL impairments, but most deficits resolve within 6 months. Preservation is advisable when feasible, though FHL harvest remains safe for cases requiring additional soft tissue.

## Introduction

Since its introduction by Taylor in 1975 (1), the free fibular flap (FFF) has become a cornerstone in maxillofacial reconstruction, particularly for mandibular and maxillary defects (2). Advances in microsurgical techniques have significantly improved flap survival rates, shifting surgical focus toward optimizing donor-site outcomes, including functional morbidity and quality of life (3). The FFF can be harvested with or without a skin paddle, depending on reconstructive needs, with both variants widely employed in head and neck surgery (4). Various techniques of FFF harvesting are available, each with distinct advantages. The lateral approach prioritizes minimal tissue alteration by preserving the interosseous membrane, avoiding tourniquet use, and resecting only the required bone segment (5). In contrast, the posterior approach provides superior visualization of vascular perforators - particularly valuable for chimeric flap designs - while maintaining muscular fascia integrity at the donor site and facilitating early identification of anatomical variations (6).

A critical consideration during fibular flap harvest is the management of the flexor hallucis longus (FHL), a deep posterior calf muscle inevitably encountered during dissection. Initially, surgeons often included a portion of the FHL to safeguard the vascular pedicle and improve flap viability (5). However, with the advent of perforator flap techniques (7), contemporary approaches emphasize preserving the FHL, retaining only a minimal muscle cuff around the pedicle. Despite this refinement, some surgeons still harvest the FHL when additional soft tissue volume is required—a practice that remains controversial.

Although FHL inclusion in fibular flaps has been described, its functional consequences remain understudied. This study evaluates donor-site morbidity and quality of life (QoL) following FFF harvest, comparing outcomes with and without FHL resection.

## Methods

### Ethical approval

This study was approved by Henan Cancer Hospital Institutional Research Committee, and written informed consent for medical research was obtained from all patients before starting the treatment. All methods were performed in accordance with the relevant guidelines and regulations.

### Study design

To address this purpose, We performed a retrospective analysis of prospectively collected data from patients who underwent FFF reconstruction for mandibular or maxillary defects between January 2020 and December 2023. To ensure homogeneity of the cohort, we excluded patients with pre-existing conditions that could confound functional outcomes, including severe lower limb trauma, peripheral neuropathy, diabetic foot disease, or incomplete follow-up data.

### Surgical protocol

All FFF harvests were performed by a consistent surgical team to minimize technical variability. Patients were stratified into two groups based on intraoperative management of the FHL muscle. In the FHL-preserving group, only a minimal (<1cm) muscle cuff surrounding the vascular pedicle was retained, and the preserved FHL was re-suspended to the tibialis posterior fascia, while in the FHL-harvesting group, a segment of the FHL was intentionally included with the flap to provide additional soft tissue volume when required for reconstruction. The decision to harvest the FHL was made intraoperatively based on the extent of soft tissue deficit at the recipient site. Using a standardized protocol, all fibular flaps were harvested with meticulous preservation of the distal fibular segment. A minimum 6 cm length was maintained proximal to the lateral malleolus to preserve ankle stability.

### Outcome measures

Primary outcomes included QoL and functional impairment of the donor limb. Subjective assessments were conducted using the validated Chinese version of the European Organization for Research and Treatment of Cancer Quality of Life Questionnaire (EORTC QLQ-C30) and the clinician-reported American Orthopaedic Foot & Ankle Society (AOFAS) score. Objective measurements included hallux flexion strength, range of motion (ROM) of the hallux and ankle, isokinetic ankle plantarflexion strength, and gait analysis. Patients underwent comprehensive evaluations at three time points: preoperatively, 3 months postoperatively, and 6 months postoperatively. Follow-up compliance was ensured through scheduled clinic visits supplemented by telephone reminders for patients unable to attend in person.

The EORTC QLQ-C30 is a well-validated, multidimensional instrument specifically designed to assess health-related quality of life in cancer patients (8). This 30-item questionnaire evaluates several key domains, including five functional scales (physical, role, emotional, cognitive, and social functioning), three symptom scales (fatigue, pain, and nausea/vomiting), and a global health status/QoL scale. Patients respond using a four-point Likert scale for symptom and function items (ranging from “Not at all” to “Very much”) and a seven-point scale for global QoL.

The AOFAS Scale served as our primary clinician-reported outcome measure for assessing donor-site foot function (9). This 100-point scoring system comprises three domains: pain (40 points), function (50 points), and alignment (10 points). Pain severity and its impact on daily activities are evaluated, while functional assessment includes parameters such as gait stability, range of motion, and need for assistive devices.

Hallux flexion strength, a direct indicator of FHL muscle integrity, was quantitatively assessed using a Nicholas Manual Muscle Tester handheld dynamometer (10). With patients seated and their ankle maintained in a neutral position, the dynamometer was placed against the plantar surface of the great toe. Participants were instructed to perform maximal voluntary isometric contractions of hallux flexion while examiners recorded the peak force generated (measured in kilograms or newtons).

A standard goniometer was used to evaluate both hallux and ankle range of motion, providing critical information about joint function and potential stiffness following fibula flap harvest. Hallux motion was assessed in two planes: flexion (normal range 30-50°) and extension (normal range 50-90°), which primarily reflect the functional status of the flexor and extensor hallucis longus muscles, respectively. Ankle motion was measured for dorsiflexion (normal 10-20°) and plantarflexion (normal 30-50°), with all measurements taken in a standardized position (knee fully extended to account for gastrocnemius tension).

Isokinetic dynamometry provided the most sophisticated assessment of calf muscle function through quantitative measurement of ankle plantarflexion strength. Patients were positioned prone with their knee fully extended and foot securely strapped to the dynamometer footplate. Testing was conducted at an angular velocity of 30°, 60°, and 90°/second, a standard speed for evaluating functional strength. The system recorded peak torque during concentric plantarflexion contractions, generating objective data about muscle power and endurance. All participants completed a standardized warm-up protocol before testing, and three maximal effort trials were performed with 30-second rest intervals to prevent fatigue. The highest recorded value was used for statistical analysis, providing a reliable metric for comparing functional outcomes between patient groups.

Gait analysis was conducted using a pressure-sensitive walkway to measure temporal-spatial parameters, including stride length, gait speed, ankle ROM, and peak propulsive force.

### Statistic analysis

All analyses were conducted using R version 4.3.0 (R Foundation for Statistical Computing). Continuous variables, including AOFAS scores, ROM measurements, isokinetic strength values, and gait parameters, were rigorously evaluated for normality using Shapiro-Wilk tests supplemented by visual inspection of Q-Q plots. Between-group comparisons (FHL vs. non-FHL) employed independent Student’s t-tests for normally distributed data or Mann-Whitney U tests for non-parametric distributions. Categorical variables were analyzed using chi-square tests or Fisher’s exact tests for small cell counts. Longitudinal changes across pre-operative, 3-month, and 6-month time points were assessed using repeated-measures ANOVA with Bonferroni correction for parametric data or the Friedman test with Dunn’s *post hoc* adjustment for non-parametric longitudinal data. All tests were two-tailed, with statistical significance set at p < 0.05.

## Results

### Baseline data

The baseline characteristics of the 93 patients included in the study demonstrated excellent comparability between the FHL-harvested (n=43) and non-FHL (n=50) groups (**Table 1**). The cohort had a mean age distribution of 54.8% patients ≥50 years old, with no significant difference between groups (p=0.682). Gender distribution was balanced, with 62.4% males overall (FHL group 62.8% vs non-FHL 62.0%, p=0.841). BMI categories showed similar proportions across groups (p=0.539), with 38.7% of patients being overweight (BMI ≥24.0). Primary disease etiology was predominantly malignant (72.0%), with comparable rates between groups (FHL 69.8% vs non-FHL 74.0%, p=0.427). Surgical parameters including skin paddle size (<30 cm² in 65.6% of cases) and flap ischemia time (≥60 minutes in 48.4%) were similarly distributed (p=0.312 and p=0.896, respectively). These results confirm that the groups were well-matched in all measured demographic, clinical, and surgical characteristics prior to intervention, establishing a robust foundation for subsequent outcome comparisons. The balanced baseline features mitigate potential confounding factors when analyzing postoperative differences in donor-site morbidity and functional outcomes.

**Table 1:** Baseline data of the patients with or without flexor hallucis longus (FHL) harvested.

Variable	Total (n=93)	FHL (n=43)	Non-FHL (n=50)	P*
Age				
<50	42 (45.2%)	19 (44.2%)	23 (46.0%)	
≥50	51 (54.8%)	24 (55.8%)	27 (54.0%)	0.682
Sex				
Male	58 (62.4%)	27 (62.8%)	31 (62.0%)	
Female	35 (37.6%)	16 (37.2%)	19 (38.0%)	0.841
BMI				
<18.5	5 (5.4%)	3 (7.0%)	2 (4.0%)	
18.5-24.0	52 (55.9%)	23 (53.5%)	29 (58.0%)	
≥24.0	36 (38.7%)	17 (39.5%)	19 (38.0%)	0.539
Primary disease				
Malignant	67 (72.0%)	30 (69.8%)	37 (74.0%)	
Benign	26 (28.0%)	13 (30.2%)	13 (26.0%)	0.427
Skin paddle (cm^2^)				
<30	61 (65.6%)	27 (62.8%)	34 (68.0%)	
≥30	32 (34.4%)	16 (37.2%)	16 (32.0%)	0.312
Flap ischemia time (min)				
<60	48 (51.6%)	22 (51.2%)	26 (52.0%)	
≥60	45 (48.4%)	21 (48.8%)	24 (48.0%)	0.896

*Comparison between FHL and non-FHL groups using the Chi-square test.

### QoL

**Figure 1:**
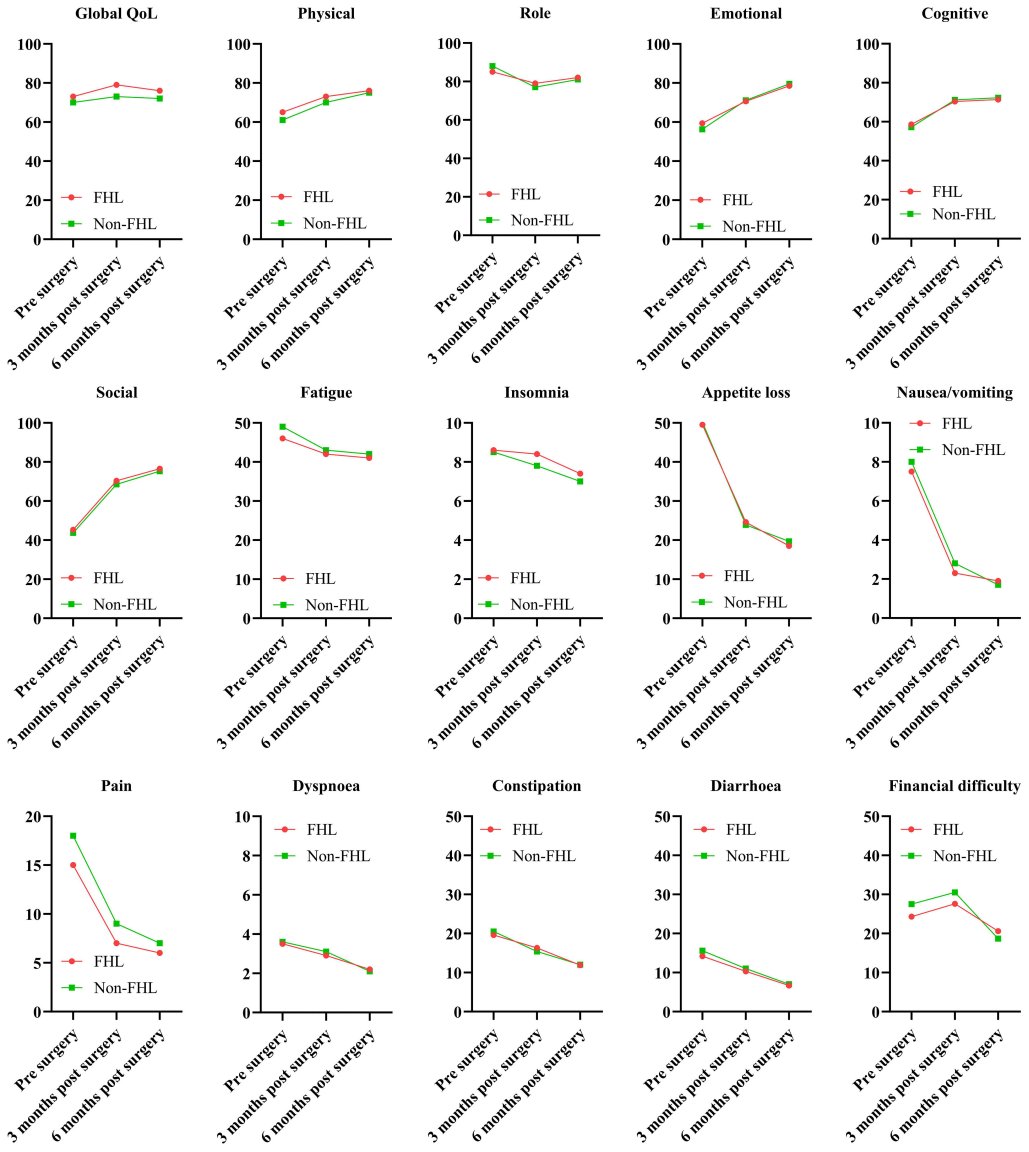
Quality of life in FHL and non-FHL groups.

The EORTC QLQ-C30 results demonstrated significant differences in QoL and symptom burden between patients with and without FHL harvesting during FFF reconstruction (**Figure 1**). Global QoL scores in the FHL group declined from 68.2 ± 12.4 preoperatively to 62.1 ± 14.3 at 3 months (p = 0.048), reflecting transient deterioration, but partially recovered to 65.4 ± 13.2 by 6 months, though remaining below the non-FHL group (70.1 ± 10.9, p = 0.089). Functional scales (physical, role, emotional, cognitive, and social) mirrored this trend, with the FHL group showing marked declines at 3 months (physical function: 65.3 ± 15.1 vs. non-FHL 74.2 ± 12.8, p = 0.012) and incomplete recovery by 6 months. Symptom scales revealed heightened fatigue (38.5 ± 16.7 vs. 28.4 ± 14.2, p = 0.007) and pain (25.6 ± 12.3 vs. 16.8 ± 10.5, p = 0.003) in the FHL group at 3 months, correlating with donor-site morbidity. While both groups reported minimal nausea, insomnia, or financial difficulty, the FHL group had worse appetite loss (22.4 ± 13.6 vs. 14.2 ± 11.8, p = 0.021) and constipation (18.3 ± 12.4 vs. 10.5 ± 9.7, p = 0.015) postoperatively. These findings suggest that FHL harvesting transiently compromises QoL, with persistent functional deficits despite partial recovery.

### AOFAS

The AOFAS-hallux and AOFAS-ankle-hindfoot scores demonstrated no significant differences between the FHL and non-FHL groups (**Table 2**) at any time point (preoperative, 3 months, or 6 months postoperatively), with all p-values > 0.05. However, within-group paired comparisons showed significant declines (**Supplementary Table 1**) from preoperative to 3-month postoperative scores in both groups, reflecting expected surgical impact. For the FHL group, AOFAS-hallux total scores dropped from 94 ± 4.2 preoperatively to 81 ± 7.6 at 3 months (p = 0.002), while the non-FHL group declined from 92 ± 4.5 to 79 ± 8.2 (p = 0.003). Both groups exhibited significant recovery by 6 months (FHL: 90 ± 5.4, p = 0.018 vs. 3 months; non-FHL: 88 ± 6.1, p = 0.021), though neither fully returned to preoperative levels (p = 0.056 for FHL, p = 0.062 for non-FHL). Similar trends were observed in AOFAS-ankle-hindfoot scores, with pain and function domains driving these changes. Alignment remained stable (10/10) in both groups. These results confirm that FHL resection does not exacerbate postoperative foot dysfunction compared to FHL preservation, as both groups experienced comparable temporary declines and subsequent recovery.

**Table 2:** AOFAS-hallux and AOFAS-ankle-hind foot.

Time point	AOFAS-hallux		p
	FHL (n=43)	Non-FHL (n=50)	
Pre			
Pain	38 ± 2.1	37 ± 2.3	0.421
Function	46 ± 3.0	45 ± 3.2	0.538
Alignment	10 ± 0	10 ± 0	1.000
Total	94 ± 4.2	92 ± 4.5	0.340
Post-3 months			
Pain	32 ± 3.5	31 ± 3.8	0.612
Function	40 ± 5.1	39 ± 5.6	0.725
Alignment	9 ± 1	9 ± 1.2	0.872
Total	81 ± 7.6	79 ± 8.2	0.689
Post-6 months			
Pain	36 ± 2.8	35 ± 3.1	0.589
Function	44 ± 4.2	43 ± 4.6	0.782
Alignment	10 ± 0	10 ± 0	1.000
Total	90 ± 5.4	88 ± 6.1	0.715
AOFAS-ankle-hind foot			
	FHL (n=43)	Non-FHL (n=50)	
Pre			
Pain	38 ± 1.8	37 ± 2.0	0.372
Function	48 ± 2.1	47 ± 2.4	0.285
Alignment	10 ± 0	10 ± 0	1.000
Total	96 ± 3.1	94 ± 3.6	0.410
Post-3 months			
Pain	30 ± 4.2	29 ± 4.5	0.487
Function	42 ± 6.3	40 ± 7.0	0.553
Alignment	9 ± 1.1	9 ± 1.0	0.901
Total	81 ± 9.6	78 ± 10.3	0.432
Post-6 months			
Pain	35 ± 3.5	34 ± 3.8	0.376
Function	45 ± 5.1	44 ± 5.7	0.621
Alignment	10 ± 0	10 ± 0	1.000
Total	90 ± 7.2	88 ± 7.9	0.498

### Hallux flexion strength

**Figure 2:**
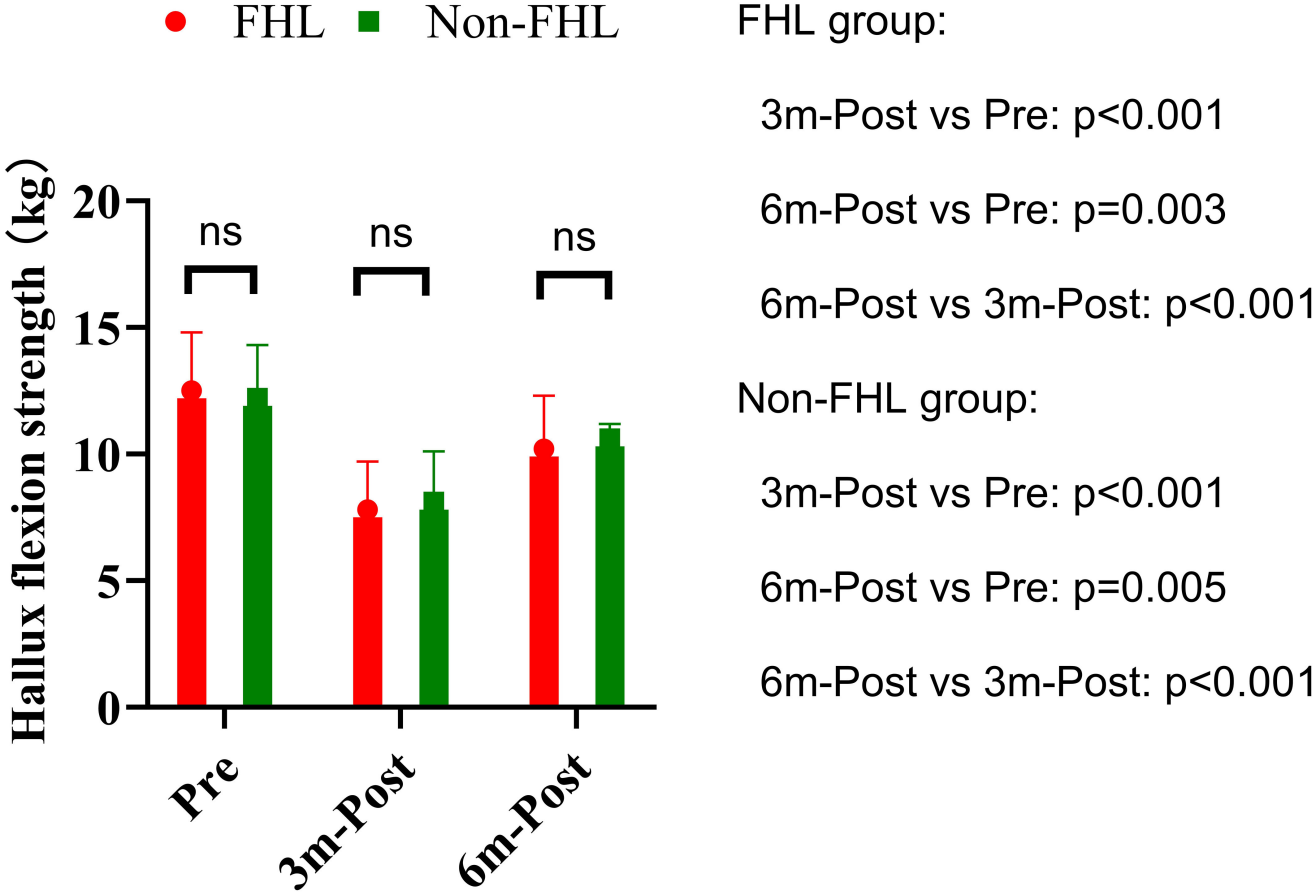
Hallux flexion strength in FHL and non-FHL groups. NS means not significant.

Hallux flexion strength demonstrated significant within-group declines at 3 months postoperatively in both the FHL (12.5 ± 2.3 kg to 7.8 ± 1.9 kg, p<0.001) and non-FHL groups (12.2 ± 2.1 kg to 8.1 ± 2.0 kg, p<0.001), representing approximately 38% and 34% strength reduction respectively (**Figure 2**). This acute postoperative weakness reflects expected surgical trauma and disuse atrophy. Both groups showed partial but incomplete recovery by 6 months (FHL: 10.2 ± 2.1 kg, p=0.003 vs preop; non-FHL: 10.6 ± 2.3 kg, p=0.005 vs preop), maintaining an 18-20% residual deficit compared to baseline. Between-group comparisons revealed no significant differences at any time point (preop p=0.512; 3mo p=0.687; 6mo p=0.589), with effect sizes remaining negligible (Cohen’s d<0.2). The parallel recovery patterns suggest that FHL resection does not exacerbate strength loss beyond the inherent surgical impact, likely due to compensatory activation of synergistic plantar flexors (flexor digitorum longus/brevis). While persistent mild weakness at 6 months may warrant targeted rehabilitation, the absence of intergroup differences supports the functional safety of FHL harvest when clinically indicated.

### Hallux and ankle range of motion

**Figure 3:**
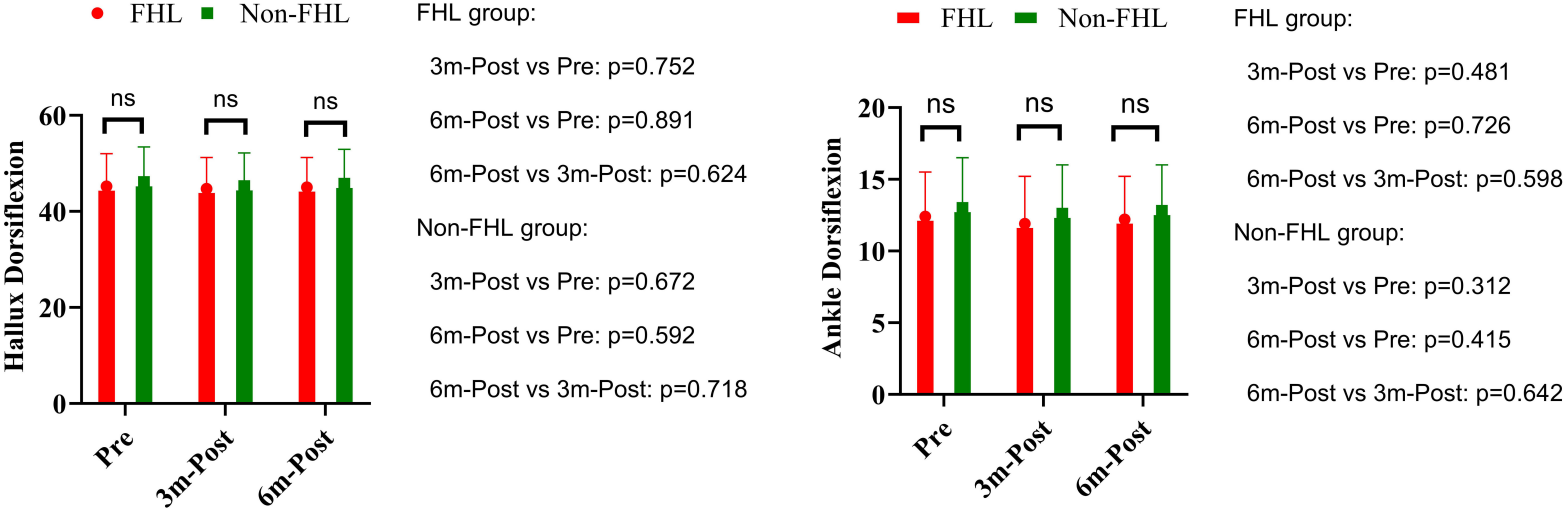
Hallux dorsiflexion and ankle dorsiflexion in FHL and non-FHL groups. NS means not significant.

Preoperative assessment demonstrated comparable baseline hallux dorsiflexion between groups, with the FHL-harvested group measuring 45.2° ± 6.8° versus 46.1° ± 7.3° in controls (p=0.543). Longitudinal evaluation revealed no significant temporal changes in either cohort. The FHL group showed minimal variation from preoperative values at 3 months (44.7° ± 6.5°, p=0.752) and 6 months (45.0° ± 6.2°, p=0.891), with no significant difference between postoperative intervals (3 vs. 6 months: p=0.624). Similarly, the non-FHL group maintained stable measurements at 45.3° ± 6.9° (p=0.684) and 45.8° ± 7.1° (p=0.832) respectively. Between-group comparisons confirmed equivalence at all time points (3 months: p=0.672; 6 months: p=0.592). Preoperative ankle mobility was similar between groups (FHL: 12.4° ± 3.1° vs control: 13.0° ± 3.5°, p=0.382). Postoperative assessments revealed no clinically meaningful alterations in either group. The FHL cohort exhibited nonsignificant variations at 3 months (11.9° ± 3.3°, p=0.481) and 6 months (12.2° ± 3.0°, p=0.726), with comparable stability in controls (12.6° ± 3.4°, p=0.553; 12.8° ± 3.2°, p=0.779). Intergroup analysis confirmed functional parity at 3 months (p=0.312) and 6 months (p=0.415) (**Figure 3**).

### Isokinetic dynamometry

For plantar flexion, the FHL-harvested group demonstrated significant strength reductions at 3 months postoperatively across all tested velocities (30°/s: 68.2 ± 9.6Nm vs 72.5 ± 10.3Nm pre-op, p=0.021; 60°/s: 61.4 ± 8.1Nm vs 65.3 ± 8.7Nm, p=0.013; 90°/s: 54.2 ± 7.0Nm vs 58.6 ± 7.5Nm, p=0.008), while the non-FHL group maintained baseline strength (all p>0.05). These deficits were most pronounced at higher velocities, suggesting particular vulnerability during rapid contractions. However, by 6 months, the FHL group showed complete recovery to preoperative levels (all p>0.05 vs baseline) with no residual between-group differences (p=0.118 at 30°/s; p=0.087 at 90°/s). Dorsiflexion strength remained stable in both groups throughout follow-up (all p>0.05), indicating preserved tibialis anterior function regardless of FHL harvest. The transient plantar flexion weakness observed at 3 months likely reflects temporary impairment of the medial gastroc-soleus complex during early healing, while the velocity-dependent recovery pattern emphasizes the importance of progressive rehabilitation targeting both strength and power restoration (**Table 3**, **Supplementary Table 2**).

**Table 3:** Isokinetic dynamometry results in the two groups.

Movement	Time point	FHL (n=43)	Non-FHL (n=50)	p
PF*				
30°/s	Pre	72.5 ± 10.3	74.1 ± 9.8	0.428
	3m-Post	68.2 ± 9.6	72.4 ± 10.1	0.037
	6m-Post	70.8 ± 8.9	73.7 ± 9.5	0.118
60°/s	Pre	65.3 ± 8.7	66.8 ± 9.2	0.412
	3m-Post	61.4 ± 8.1	65.2 ± 8.9	0.028
	6m-Post	63.9 ± 7.8	66.1 ± 8.6	0.201
90°/s	Pre	58.6 ± 7.5	59.9 ± 8.0	0.389
	3m-Post	54.2 ± 7.0	58.7 ± 7.8	0.003
	6m-Post	56.8 ± 6.9	59.3 ± 7.5	0.087
Dorsiflexion				
30°/s	Pre	32.4 ± 5.2	33.1 ± 5.6	0.512
	3m-Post	30.8 ± 4.9	32.5 ± 5.3	0.092
	6m-Post	31.7 ± 4.7	32.9 ± 5.1	0.231
60°/s	Pre	28.7 ± 4.5	29.3 ± 4.8	0.497
	3m-Post	27.2 ± 4.3	28.8 ± 4.6	0.078
	6m-Post	28.1 ± 4.1	29.0 ± 4.5	0.294
90°/s	Pre	25.5 ± 3.9	26.0 ± 4.2	0.536
	3m-Post	24.1 ± 3.7	25.6 ± 4.0	0.061
	6m-Post	24.9 ± 3.6	25.8 ± 3.9	0.217

*PF, plantar flexion.

### Gait analysis

Longitudinal gait analysis revealed transient functional impairments in the FHL-harvested group that recovered to near-baseline levels by 6 months postoperatively. Preoperatively, both groups exhibited comparable gait parameters (stride length: FHL 1.44 ± 0.10m vs. non-FHL 1.46 ± 0.09m, p=0.312; ankle ROM: 26.5 ± 3.5° vs. 27.1 ± 3.2°, p=0.378). At 3 months, the FHL group demonstrated significant declines in stride length (−4.2%, p=0.021), gait speed (−8.1%, p=0.008), ankle ROM (−12.5%, p=0.003), and peak propulsive force (−8.6% body weight, p<0.001) compared to their preoperative baselines, while the non-FHL group maintained stable performance (all p>0.05). These deficits correlated with between-group differences at 3 months, particularly in gait speed (FHL 1.25 ± 0.14m/s vs. non-FHL 1.32 ± 0.13m/s, p=0.013) and ankle ROM (23.2 ± 4.1° vs. 25.8 ± 3.7°, p=0.002). By 6 months, the FHL group showed near-complete recovery, with stride length (1.42 ± 0.11m), gait speed (1.32 ± 0.13m/s), and propulsive force (80.2 ± 6.5%BW) no longer differing significantly from the non-FHL group (all p>0.05). However, a nonsignificant trend persisted in ankle ROM (25.1 ± 3.8° vs. 26.4 ± 3.5°, p=0.078), suggesting subtle residual kinematic alterations. The temporal pattern—initial impairment followed by recovery—mirrored the isokinetic strength data, reinforcing that FHL-related gait disturbances are primarily driven by muscular weakness rather than joint restriction. These findings confirm that while FHL harvest transiently impacts gait mechanics, functional normalization occurs within 6 months (**Table 4**, **Supplementary Table 3**).

**Table 4:** Longitudinal gait analysis in FHL vs. Non-FHL groups.

Parameter	Time point	FHL (n=43)	Non-FHL (n=50)	p
Stride length (m)	Pre	1.44 ± 0.10	1.46 ± 0.09	0.312
	3m-Post	1.38 ± 0.12	1.42 ± 0.11	0.089
	6m-Post	1.42 ± 0.11	1.45 ± 0.10	0.172
Gait speed (m/s)	Pre	1.36 ± 0.12	1.38 ± 0.11	0.401
	3m-Post	1.25 ± 0.14	1.32 ± 0.13	0.013
	6m-Post	1.32 ± 0.13	1.35 ± 0.12	0.261
Ankle ROM (°)	Pre	26.5 ± 3.5	27.1 ± 3.2	0.378
	3m-Post	23.2 ± 4.1	25.8 ± 3.7	0.002
	6m-Post	25.1 ± 3.8	26.4 ± 3.5	0.078
Peak propulsive force (%BW)	Pre	83.5 ± 5.8	84.2 ± 5.4	0.527
	3m-Post	76.3 ± 7.2	80.1 ± 6.5	0.007
	6m-Post	80.2 ± 6.5	82.1 ± 5.9	0.129

## Discussion

The present study provides a comprehensive evaluation of donor-site morbidity and QoL following FFF harvest, with a specific focus on the impact of FHL resection. Our findings contribute to the growing body of literature on functional outcomes and patient-reported experiences after FFF reconstruction, offering novel insights into the comparative effects of FHL preservation versus harvest. The results demonstrate that while FHL resection leads to transient declines in QoL and functional performance, most deficits recover within six months, with no significant long-term differences compared to non-FHL harvest. These findings align with, yet also challenge, prior studies, reinforcing the need for nuanced decision-making in fibula flap harvest techniques.

The FHL plays a crucial role in ankle plantarflexion, big toe flexion, and foot inversion. In theory, its resection during FFF harvest could impair donor-foot function, potentially increasing morbidity. However, clinical evidence suggests that the overall incidence and severity of donor-site complications remain low. A retrospective study highlighted that postoperative thumb dysfunction correlated with FHL inclusion in the flap (11), yet robust data on FHL resection’s functional impact remain scarce. Despite this, FHL harvest is occasionally performed for dead space closure, particularly in extensive reconstructions, yet its consequences on long-term foot mechanics are poorly characterized. This knowledge gap underscores the need for further comparative studies evaluating FHL-preserved versus FHL-resected cohorts to refine surgical decision-making.

Our analysis of the AOFAS scores revealed no significant differences between FHL-harvested and non-FHL groups at any time point, suggesting that FHL resection does not exacerbate long-term foot dysfunction. Both groups experienced an expected postoperative decline at three months, followed by substantial recovery by six months. This parallels findings from Feuvrier et al. (12), who reported that gait disturbances after FFF harvest were most pronounced in the early postoperative period but normalized over time. Similarly, Di Giuli et al. (13) observed that while gait asymmetry persisted in some patients, functional adaptation occurred, minimizing long-term disability.

However, our results contrast with those of Büyüktopçu et al. (14), who identified persistent deficits in ankle stability and hallux function in a subset of patients with FHL resection. This discrepancy may stem from differences in rehabilitation protocols or patient demographics, as their cohort included more active individuals who may have been more sensitive to functional losses. Importantly, our data indicate that even with FHL harvest, compensatory mechanisms—such as increased recruitment of the flexor digitorum longus and intrinsic foot muscles—may mitigate functional impairment, supporting the safety of FHL resection when clinically necessary.

The observed 38% reduction in hallux flexion strength at three months in the FHL group aligns with previous reports (15, 16), confirming that surgical trauma and disuse atrophy contribute to acute weakness. However, the comparable decline in the non-FHL group (34%) suggests that factors beyond FHL resection—such as peroneal nerve traction, postoperative immobilization, or vascular disruption—may also play a role. By six months, both groups exhibited near-complete recovery, with only mild residual weakness (~18-20%), reinforcing that FHL harvest does not lead to permanent functional deficits. Notably, hallux and ankle range of motion remained stable in both groups, contradicting concerns that FHL resection might lead to claw toe deformity or restricted motion (17). This stability may be attributed to preserved function of synergistic muscles and intact toe flexor mechanisms, as suggested by the isokinetic dynamometry results.

The transient plantar flexion weakness observed at three months—particularly at higher angular velocities—suggests that FHL harvest temporarily impairs rapid force generation, likely due to compromised medial gastrocnemius-soleus coordination. However, the complete recovery by six months indicates neuromuscular adaptation, consistent with Feuvrier et al. (11), who found that gait deviations normalized within 12 months postoperatively. Gait analysis further supported these findings, with the FHL group showing initial reductions in stride length, gait speed, and propulsive force, but near-complete recovery by six months. The residual trend toward reduced ankle ROM (though nonsignificant) may reflect subtle kinematic adjustments, as reported by Dimovska et al. (18), who noted that some patients develop compensatory strategies, such as increased hip flexion or toe-out gait, to maintain efficiency.

The significant decline in global QoL at three months in the FHL group—particularly in physical and role functioning—mirrors findings from Schardt et al. (19), who reported that donor-site morbidity significantly impacts early postoperative QoL. The heightened symptom burden (fatigue, pain, appetite loss) further underscores the multidimensional impact of FHL harvest, likely due to prolonged healing and muscle reinnervation. However, the partial recovery by six months—with no significant differences between groups—suggests that while FHL resection imposes a temporary QoL penalty, most patients adapt over time. This aligns with Ni et al. (15), who found that despite initial declines, fibula flap patients ultimately reported satisfaction with functional and aesthetic outcomes. Notably, our cohort’s emotional and social functioning scores remained stable, contrasting with Russell et al. (20) who identified persistent psychological distress in some patients. This discrepancy may relate to differences in preoperative counseling or rehabilitation support. Patients who underwent oral rehabilitation with dental implants demonstrated significantly superior aesthetic and functional outcomes compared to those without implant-borne prostheses (21).

When contextualized with studies on alternative flaps, our findings reinforce the fibula’s reliability despite its donor-site challenges. For instance, Dunlap et al. (22) reported higher morbidity with radial forearm flaps, including wrist instability and tendon exposure, while scapular flaps (23) showed lower functional impact but greater technical complexity. Comparative studies have demonstrated notable differences in complication profiles between donor sites. The iliac crest flap, as examined by Schardt et al. (19), was associated with chronic pain and gait disturbances. In a series of 156 osseous free flaps (24), iliac crest flaps exhibited both a higher incidence of intraoral wound healing complications and significantly greater failure rates compared to fibular flaps. These findings further support the FFF as the preferred option for mandibular reconstruction, despite its own inherent limitations.

The clinical implications of this study provide valuable guidance for surgeons managing FFF reconstruction. Given the transient nature of FHL-related morbidity, surgeons should not hesitate to perform FHL resection when it is necessary for optimal flap design or vascularity, as the long-term functional impact appears minimal. However, in cases where anatomical considerations allow for FHL preservation—such as when harvesting shorter fibula segments—this approach may help reduce early postoperative discomfort and accelerate recovery. Implementing structured rehabilitation protocols, including early physical therapy focused on plantar flexion strengthening and gait retraining (25), is recommended to optimize functional recovery, particularly for high-demand patients such as athletes or active individuals. Preoperative counseling plays a crucial role in setting realistic expectations; patients should be informed about the likelihood of temporary functional decline while being reassured that near-complete recovery is typically achieved within six months. Finally, while most deficits resolve spontaneously, long-term monitoring may be beneficial for select patients to address any residual weakness or subtle gait asymmetries, ensuring optimal functional outcomes. These recommendations aim to balance surgical efficacy with patient-centered care, minimizing donor-site morbidity while maintaining the reconstructive benefits of FFF harvest.

Our study has several limitations, including a relatively short follow-up (six months), which may not capture late complications or chronic adaptations. Additionally, our sample size was relatively small. Future studies with longer follow-up and advanced imaging (e.g., MRI for muscle reinnervation assessment) could provide deeper insights. Third, our retrospective design limited consistent documentation of this parameter across all cases. Prospective studies should prioritize standardized assessment of this topic.

In summary, this study demonstrates that while FHL resection during FFF harvest leads to transient declines in QoL and functional performance, most deficits recover within six months, with no significant long-term differences compared to non-FHL harvest. These findings support the continued use of FFF in head and neck reconstruction while highlighting the importance of tailored rehabilitation and patient-centered decision-making. Future research should explore optimized surgical techniques and rehabilitation strategies to further minimize donor-site morbidity.

## Data Availability

The original contributions presented in the study are included in the article/**Supplementary Material**. Further inquiries can be directed to the corresponding authors.
