# Supplementary material for: Functional outcomes and quality of life following free fibula flap harvest: a comparative analysis of flexor hallucis longus resection versus preservation
Source: Front Oncol. 2025 Sep 5;15:1651547. doi: 10.3389/fonc.2025.1651547 (PMC12446026; doi:10.3389/fonc.2025.1651547)
Supplement: Supplementary file 1 [file Table1.doc]

Supplementary Table 1. Pair test at different time points in FHL and non-FHL groups.

| Domain | Group | Pre vs 3m-Post | 3m-Post vs 6m-Post | Pre vs 6m-Post |
| --- | --- | --- | --- | --- |
| Pain | FHL | 0.001 | 0.004 | 0.072 |
|  | Non-FHL | 0.002 | 0.006 | 0.085 |
| Function | FHL | 0.003 | 0.010 | 0.048 |
|  | Non-FHL | 0.004 | 0.015 | 0.053 |
| Alignment | FHL | 0.157 | 0.210 | 1.000 |
|  | Non-FHL | 0.182 | 0.195 | 1.000 |
