# Supplementary material for: Functional outcomes and quality of life following free fibula flap harvest: a comparative analysis of flexor hallucis longus resection versus preservation
Source: Front Oncol. 2025 Sep 5;15:1651547. doi: 10.3389/fonc.2025.1651547 (PMC12446026; doi:10.3389/fonc.2025.1651547)
Supplement: Supplementary file 2 [file Table2.doc]

Supplementary Table 2. Pair test at different time points in FHL and non-FHL groups.

| Movement | Group | Pre vs 3m-Post | Pre vs 6m-Post | 3m-Post vs 6m-Post |
| --- | --- | --- | --- | --- |
| Plantar flexion |  |  |  |  |
| 30°/s | FHL | 0.021 | 0.342 | 0.018 |
|  | Non-FHL | 0.157 | 0.491 | 0.284 |
| 60°/s | FHL | 0.013 | 0.265 | 0.009 |
|  | Non-FHL | 0.208 | 0.537 | 0.326 |
| 90°/s | FHL | 0.008 | 0.178 | 0.006 |
|  | Non-FHL | 0.241 | 0.603 | 0.419 |
| Dorsiflexion |  |  |  |  |
| 30°/s | FHL | 0.104 | 0.423 | 0.087 |
|  | Non-FHL | 0.276 | 0.588 | 0.354 |
| 60°/s | FHL | 0.087 | 0.376 | 0.102 |
|  | Non-FHL | 0.312 | 0.624 | 0.401 |
| 90°/s | FHL | 0.065 | 0.312 | 0.079 |
|  | Non-FHL | 0.358 | 0.712 | 0.442 |
