# Supplementary material for: Functional outcomes and quality of life following free fibula flap harvest: a comparative analysis of flexor hallucis longus resection versus preservation
Source: Front Oncol. 2025 Sep 5;15:1651547. doi: 10.3389/fonc.2025.1651547 (PMC12446026; doi:10.3389/fonc.2025.1651547)
Supplement: Supplementary file 3 [file Table3.doc]

Supplementary Table 3. Pair test at different time points in FHL and non-FHL groups.

| Parameter | Group | Pre vs 3m-Post | Pre vs 6m-Post | 3m-Post vs 6m-Post |
| --- | --- | --- | --- | --- |
| Stride length | FHL | 0.012 | 0.214 | 0.009 |
|  | Non-FHL | 0.087 | 0.342 | 0.154 |
| Gait speed | FHL | 0.003 | 0.127 | 0.005 |
|  | Non-FHL | 0.112 | 0.285 | 0.203 |
| Ankle ROM | FHL | 0.001 | 0.038 | 0.004 |
|  | Non-FHL | 0.059 | 0.421 | 0.076 |
| Peak propulsive force | FHL | <0.001 | 0.021 | 0.002 |
|  | Non-FHL | 0.134 | 0.387 | 0.218 |
